# Infant mortality in the Metropolitan Region of São Paulo: an ecological study

**DOI:** 10.31744/einstein_journal/2021AO5663

**Published:** 2021-08-02

**Authors:** Michele Ribeiro Alexandre Nunes, Luiz Vinicius de Alcantara Sousa, Vânia Barbosa do Nascimento

**Affiliations:** 1 Centro Universitário FMABC Santo AndréSP Brazil Centro Universitário FMABC, Santo André, SP, Brazil.

**Keywords:** Infant mortality, Pregnancy, Risk factors, Educational status, Maternal age

## Abstract

**Objective:**

To determine the impact of risk factors on infant mortality in the Metropolitan Region of São Paulo according to maternal and neonate characteristics, as well as mode of delivery.

**Methods:**

An ecological, quantitative study based on secondary data retrieved from infant mortality and live birth data systems. Data from 39 municipalities located in the Metropolitan Region of São Paulo were analyzed. Newborn and maternal variables were extracted from the Information Technology Department of the Unified Health System. Absolute and relative frequencies were presented, as well as linear regression and Pearson´s correlation coefficient.

**Results:**

The following maternal profile prevailed from 2006 to 2016: 8 to 11 years of education (β=73.58; p=0.023), age between 30 and 34 years (β=19.04; p=0.015) and delivery by cesarean section (β=39.59; p=0.009) after full-term pregnancy (β=-14.20; p=0.324). Mortality rates decreased in neonates compared to other age groups (β=-25.30; p<0.001). Infant mortality rates tended to be higher among women experiencing pre-term (r=0.86; p<0.001) or post-term (r=0.95; p<0.001) gestation.

**Conclusion:**

Maternal age and level of education increased among women giving birth in the Metropolitan Region of São Paulo from 2006 to 2016. These were relevant factors for infant mortality rate reduction.

## INTRODUCTION

The analysis of infant mortality rates (IMR) and related elements contributes to the understanding of living conditions in the first year of life, since risk factors for infant mortality vary during of the prenatal period, childbirth and puerperium. Infant mortality rates can be divided into neonatal (early or late) and post-neonatal mortality.^( 1 )^

Infant mortality is associated with socioeconomic, behavioral and biological factors.^( 2 )^ Neonatal mortality is vulnerable to conditions inherent to gestation and childbirth, and to genetic problems, fetal malformations and delivery-related or postpartum complications, which are more complex from a preventive perspective.^( 3 )^

Infant mortality is also influenced by external factors associated with death in this age group, such as maternal characteristics and living conditions, including environment, nutritional, socioeconomic and educational factors, access to healthcare and access to wellness services.^( 4 )^

Hence, the causes of infant mortality may indicate inappropriate application of known preventive actions.^( 5 )^

In the last decades, Brazil saw a significant drop in late infant mortality, with lower neonatal mortality and higher pre-term birth rates.^( 6 )^

According to the 2010 census of the Brazilian Institute of Geography and Statistics (IBGE - *Instituto Brasileiro de Geografia e Estatística* ), mortality rates of infants aged under 1 year declined by 47.6%, from 2000 to 2010.^( 7 )^

Infant mortality reduction is included in the Millennium Development Goals (MDGs). Brazil is making progress in that area, but has yet to reduce these deaths before 2030.^( 8 )^

The Metropolitan Region of São Paulo (RMSP - *Região Metropolitana de São Paulo* ) is an important Brazilian region due to its high population density, social inequality and urban complexities. Public policies implemented in the last decades, especially health policies, must rely on data and indicators to effectively inform programs and actions aimed at better quality of life. Delineation of infant mortality profiles may subsidize public policy monitoring and assessment.

In Brazil, the refinement of health information systems, particularly the Mortality Information System (SIM - *Sistema de Informações sobre Mortalidade* ) and the Liveborn Information System (SINASC - *Sistema de Informações sobre Nascidos Vivos* ), has led to improvements in the quality and dissemination of data on infant mortality and its determining factors.^( 9 )^

## OBJECTIVE

To determine the impact of risk factors on infant mortality in the Metropolitan Region of São Paulo according to maternal and neonate characteristics, as well as mode of delivery.

## METHODS

### Study design

An observational, ecological, quantitative study based on secondary data on infant mortality.^( 10 )^

Infant mortality data collected from 2006 to 2016 were analyzed.

### Setting

The 39 municipalities forming the RMSP are located in the vicinity of the city of São Paulo, in the Brazilian Southeast. These municipalities have the highest urban population density in the country.^( 11 )^

The RMSP was created in 1973 and restructured in 2011 by the complementary law No. 1.13916, dated June 2011. This law determined the creation of sub-regions, as published in the website (https://www.pdui.sp.gov.br/rmsp/?page_id=56).^( 12 )^ The MRSP includes the municipality of São Paulo and the following sub-regions: North (Caieiras, Cajamar, Francisco Morato, Franco da Rocha and Mairiporã), East (Arujá, Biritiba-Mirim, Ferraz de Vasconcelos, Guararema, Guarulhos, Itaquaquecetuba, Mogi das Cruzes, Poá, Salesópolis, Santa Isabel and Suzano), Southeast (Diadema, Mauá, Ribeirão Pires, Rio Grande da Serra, Santo André, São Bernardo do Campo and São Caetano do Sul), Southwest (Cotia, Embu das Artes, Embu-Guaçu, Itapecerica da Serra, Juquitiba, São Lourenço da Serra, Taboão da Serra and Vargem Grande Paulista) and West (Barueri, Carapicuíba, Itapevi, Jandira, Osasco, Pirapora do Bom Jesus and Santana do Parnaíba).

This region comprises approximately 21.6 million inhabitants (IBGE, 2018).

### Data collection

Data were extracted from the website of the Information Technology Department of the Unified Health System (DATASUS - *Departamento de Informática do Sistema Único de Saúde* ) using the TABNET tool, which provides data on Brazilian health.

DATASUS systems employed for data collection were SINASC and SIM.^( 13 )^SINASC was created by the Ministry of Health, in 1990, for systematic recording of live births in the country based on data obtained from Liveborn Certificate, which includes maternal, prenatal, delivery and newborn information.^( 14 )^ SIM was created in 1975 and comprises data on death, extracted from Death Certificate, a standardized form.^( 15 )^

Liveborn data analysis was carried out according to SINACS groups ( *i.e.* , maternal, neonate and gestation characteristics). The following maternal characteristics were analyzed: age (organized by age groups: 20-24, 25-29, 30-34, 35-39, 40-44, 45-49 and 50-54 years), mode of delivery (cesarean section or vaginal), level of education (1 to 3 years, 4 to 7 years, 8 to 11 years, and 12 or more years of study) and gestational age (under 22, 22-27, 28-31, 33-36, 37-41, and 42 weeks or longer). Neonatal data were sex (male or female) and estimated number of deaths of infants aged less than 1 year in the RMSP, between 2006 and 2016.

Data extracted from SIM were as follows: maternal characteristics such as age (20-24, 25-29, 30-34, 35-39, 40-44, 45-49, and 50-54 years), mode of delivery (cesarean section or vaginal), level of education (1 to 3 years, 4 to 7 years, 8 to 11 years, and 12 or more years of study) and gestational age (under 22, 22-27, 28-31, 33-36, 37-41, and 42 weeks or longer). Infant deaths were categorized according to age group (zero to 6, 7 to 27 and 28 to 364 days) and sex (male or female).

Early neonatal (zero to 6 days of life), neonatal (7 to 27 days of life), post-natal (28 to 364 days of life) and infant (before 1 year of age) mortality rates were calculated per 1,000 live births.

### Statistical analysis

Infant mortality trends were examined using linear regression models. Infant mortality rate and time expressed in years equivalent to the experimental period (2006 to 2016) were used as dependent and independent variables respectively.

The level of confidence was set at 95%. Analyses were conducted using software (Stata, version 11.0^®^).

### Ethics committee

This study was based on secondary data. Hence, individuals cannot be identified. Data are available on the Internet for free and unrestricted access. Therefore, this project was exempt from submission to the Research Ethics Committee, as provided by Resolution 466/2012.

## RESULTS

Analysis of maternal characteristics associated with births recorded in the RMSP, between 2006 and 2016 ( Table 1 ), revealed a significant increase in maternal levels of education, particularly in the groups from 8 to 11 years (β=73.58; p=0.023) and 12 years or more (β=15.33. p=0.024) of study.

**Table 1:** Analysis of maternal characteristics in the Metropolitan Region of São Paulo

Variable	β	r	p value*
Maternal level of education, years			
1-3	-10.66	0.79	<0.001
4-7	-22.30	0.38	0.025
8-11	73.58	0.39	0.023
12 or more	15.33	0.38	0.024
Maternal age, years			
10-14	-0.07	-0.10	0.89
15-19	0.87	-0.10	0.89
20-24	-6.12	-0.05	0.495
25-29	8.03	0.01	0.301
30-34	19.04	0.43	0.015
35-39	16.26	0.71	<0.001
40-44	5.47	0.43	0.016
45-49	0.836	0.37	0.026
50-54	-0.006	-0.16	0.912
Mode of delivery			
Vaginal	-12.27	-0.03	0.437
Cesarean section	39.59	0.49	0.009
Gestation, weeks			
<22	8.92	0.54	0.005
22-27	11.2	0.19	0.097
28-31	-12.24	0.68	<0.001
33-36	-7.25	0.06	0.229
37-41	14.20	0.01	0.323
42 or more	-3.45	0.69	0.001

Source: Brasil. Ministério da Saúde. Departamento de Informática do Sistema Único de Saúde do Brasil (DATASUS).

SIM – Sistema de Informações de Mortalidade. Brasília (DF): DATASUS; 2008 [citado 2020 Abr 6]. Disponível em: http://www2.datasus.gov.br/DATASUS/index.php?area=060701 ^(16)^

* linear regression.

β: regression slope; r: predictive capacity.

Mothers aged 30 to 34 (β=19.04; p=0.015) or 35 to 39 (β=16.26; p≤0.001) years prevailed in this sample. The number of cesarean sections (β=39.59; p=0.009) increased over the years relative to vaginal deliveries (β=-12.27; p=0.437), as did the number of full-term pregnancies (37 to 41 weeks, β=14.20; p=0.324).

**Figure 1:**
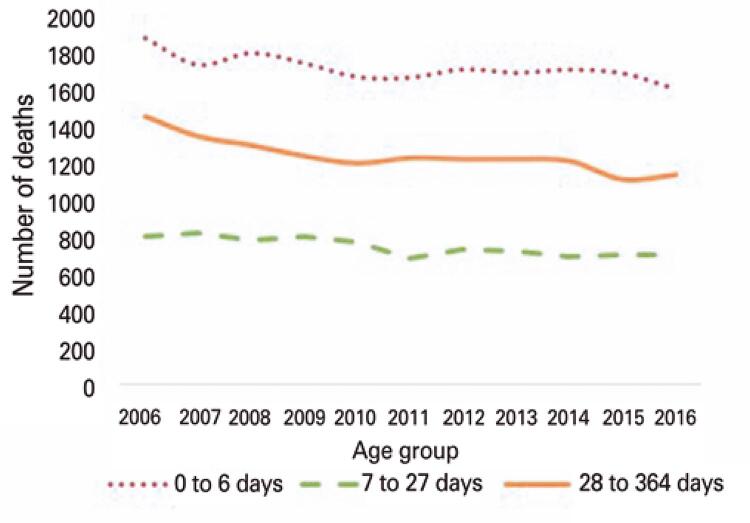
Temporal trends in infant mortality rates

Figure 1 illustrates the decline of infant mortality rates per age group in the RMSP from 2006 to 2016. Data shown in table 2 reveal a significant drop in post-neonatal (28 to 364 days, β=-25.30; p<0.001) as well as in early neonatal (zero to 6 days, β=-17.60; p=0.004) mortality rates. Mortality rates did not differ significantly according to sex.

**Table 2:** Analysis of characteristics associated with neonatal mortality in the Metropolitan Region of São Paulo

Variable	β	r	p value*
Age group, days			
0-6	-17.60	0.56	0.004
7-27	-13.62	0.71	<0.001
28-364	-25.30	0.76	<0.001
Sex			
Male	321.15	0.07	0.207
Female	341.63	0.13	0.144

Source: Brasil. Ministério da Saúde. Departamento de Informática do Sistema Único de Saúde do Brasil (DATASUS).

SIM – Sistema de Informações de Mortalidade. Brasília (DF): DATASUS; 2008 [citado 2020 Abr 6]. Disponível em: http://www2.datasus.gov.br/DATASUS/index.php?area=060701 ^(16)^

* linear regression.

β: regression slope; r: predictive capacity.

**Figure 2:**
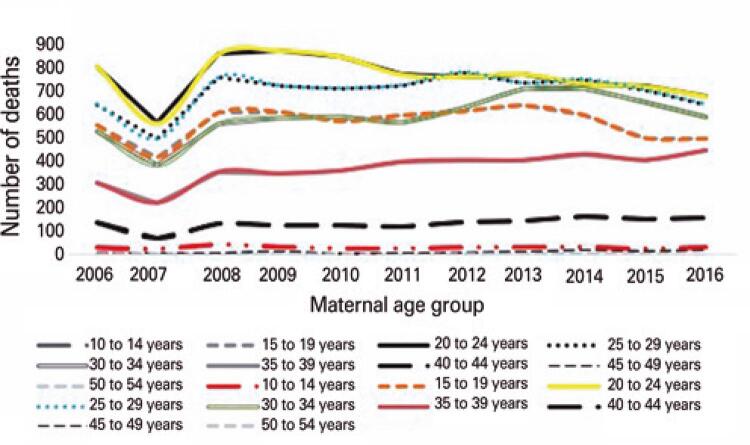
Infant mortality trends according to maternal age

Figure 2 illustrates infant mortality trends according to maternal age. Infant mortality tended to decline, with a more significant drop in the year of 2007 across all maternal age groups. Infant mortality rates increased significantly in the age groups 20-24 years, in 2008, and in the 30-34 years, in 2013.

Data presented in table 3 show higher rates of cesarean sections among women with higher levels of education – 8 to 11 years (r=0.97; p<0.001) or 12 years or more of study (r=0.91; p<0.001), and among those aged 30-34 or 35-39 years (r=0.95; p<0.001 and r=0.92; p<0.001, respectively). Vaginal delivery prevailed among women with low levels of education (4 to 7 years; r=0.85; p<0.001) and aged 20-24 years (r=0.98; p<0.001).

**Table 3:** Correlation between level of education, maternal age and mode of delivery in the Metropolitan Region of São Paulo

Variable	Vaginal		Cesarean	
	r*	p value*	r*	p value*
Maternal level of education, years				
1-3	0.46	0.146	0.54	0.080
4-7	0.85	<0.001	-0.08	0.810
8-11	0.45	0.159	0.97	<0.001
12 or more	0.48	0.128	0.91	<0.001
Maternal age group, years				
10-14	0.58	0.059	0.42	0.191
15-19	0.85	<0.001	0.66	0.025
20-24	0.98	<0.001	0.35	0.278
25-29	0.74	0.008	0.85	<0.001
30-34	0.38	0.239	0.95	<0.001
35-39	0.21	0.528	0.92	<0.001
40-44	0.30	0.357	0.85	<0.001
45-49	-0.23	0.491	0.46	0.147
50-54	-0.71	0.045	-0.24	0.555

Source: Brasil. Ministério da Saúde. Departamento de Informática do Sistema Único de Saúde do Brasil (DATASUS).

SIM – Sistema de Informações de Mortalidade. Brasília (DF): DATASUS; 2008 [citado 2020 Abr 6]. Disponível em: http://www2.datasus.gov.br/DATASUS/index.php?area=060701 ^(16)^

* Pearson’s correlation test.

**Figure 3:**
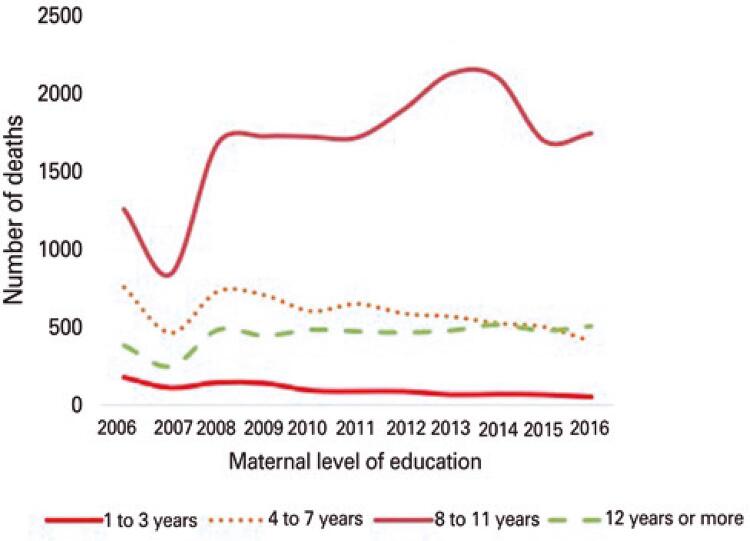
Infant mortality trends according to maternal level of education

Figure 3 illustrates infant mortality trends according to maternal level of education in the RMSP between 2006 and 2016.

Data in table 4 reveal higher infant mortality rates among pre-term (28 to 31 weeks; r=0.86, p<0.001) or post-term (42 or more; r=0.95, p<0.001) child birth. As to maternal age, this analysis revealed a significant decline in IMR among mothers aged 35-39 years (r=-0.81; p<0.002), from 2006 to 2016. Maternal level of education was associated with higher infant mortality among mothers with 1 to 3 years of study (r=0.89; p<0.001).

**Table 4:** Gestational age, maternal age and maternal level of education according to infant mortality in the Metropolitan Region of São Paulo

Variable	Infant mortality rate		
		r	p value*
Gestational age, weeks			
22-27	-0.46		0.149
28-31	0.86		<0.001
32-36	0.38		0.244
37-41	-0.41		0.205
42 or more	0.95		<0.001
Maternal age, years			
10-14	0.14		0.661
15-19	-0.10		0.757
20-24	0.13		0.696
25-29	-0.43		0.183
30-34	-0.65		0.027
35-39	-0.81		0.002
40-44	-0.55		0.077
45-49	-0.43		0.185
50-54	0.08		0.836
Maternal level of education, years			
1-3	0.89		<0.001
4-7	0.56		0.067
8-11	-0.66		0.024
12 or more	-0.67		0.022

Source: Brasil. Ministério da Saúde. Departamento de Informática do Sistema Único de Saúde do Brasil (DATASUS).

SIM – Sistema de Informações de Mortalidade. Brasília (DF): DATASUS; 2008 [citado 2020 Abr 6]. Disponível em: http://www2.datasus.gov.br/DATASUS/index.php?area=060701 ^(16)^

* Pearson’s correlation test.

## DISCUSSION

This study was based on deliveries recorded in the RMSP. The analysis of maternal profile and characteristics revealed women living in this region had high levels of education (more than 8 years of study) and were aged 30-39 years in most cases. Full-term child birth and delivery by cesarean section also prevailed in this sample. However, low levels of maternal education (1 to 3 years of study) are thought to have negative impacts on infant mortality, since this variable is often used as an indicator of maternal and family socioeconomic status, and is associated with the quality of child health care.^( 17 , 18 )^ Maternal level of education impacts infant mortality and lack of maternal education is directly associated with higher risk of infant mortality.^( 17 , 18 )^ Records also show that, from 2001 to 2011 (relative risk of 4.89 and 5.06, respectively), and from 2000 to 2003, women with 3 years of education or less had 1.56-fold higher chances of giving birth to a child.

In a study conducted in the Metropolitan Region of Porto Alegre from 1998 to 2006, maternal level of education had positive impacts on IMR, since the rate of women with 8 years or more of education increased from 46.09% in 1996 to 60.98%, in 2008.^( 19 )^

However, other studies reported that 43.2% of mothers with high levels of education (more than 8 years of study) were more associated with infant mortality.^( 20 )^

Research data revealed maternal age between 35 and 39 years is a protective factor against infant mortality. Findings reported by Alberto et al.,^( 21 )^ showed that, in Mozambique, older maternal age is also a protective factor, whereas adolescent mothers are associated with infant mortality. Data from Brazil and Mozambique suggest mature mothers manage gestation better, since they tend to undergo prenatal care and are better at infant care provision.

A study conducted in the city of Londrina (PR), from 2000 to 2009, revealed higher infant mortality rates among women aged 10 to 19 years across almost all biennia. Infant mortality rates were also higher among adolescents relative to mothers with advanced reproductive age.^( 22 )^

Prematurity is the most common risk factor for infant mortality in reported in literature. In this study, most infant deaths were associated with gestational age of 28-31 weeks. Likewise, Sanders et al.,^( 23 )^ reported higher odds of infant death among mothers giving birth with less than 37 weeks of gestation (confidence interval 6.3-66.8; p<0.001).

As to factors associated with infant mortality, this study revealed significantly lower IMR among neonates aged over 28 days. The early period of neonatal life, the first 24 hours of life in particular, is the primary determining factor of infant mortality (57.1%).^( 8 )^

Correlations between mode of delivery and maternal level of education in this study revealed higher rates on cesarean section among women with 12 years of education or more. Riscado et al.,^( 24 )^ also reported higher rates of cesarean section among women with higher socioeconomic status, higher levels of education, and access to private health care, suggesting this procedure is influenced by market trends.

Health professionals must be duly informed about modes of delivery and risks associated with elective cesarian section and iatrogenic prematurity.^( 5 )^ Cesarean section may be a protective factor against infant mortality, particularly in high-risk gestations.^( 23 )^

The relation between mode of delivery and maternal age is a controversial topic, since adolescents are arguably not prepared to withstand vaginal delivery due to lack of bodily maturity or emotional frailty. However, the obstetric development of adolescents is thought to be equivalent to that of adult women with regard to mode of delivery. Cesarian section rates are on the rise due to increasing maternal age, since more mature women who started reproduction later and have planned gestations are candidates for elective cesarian section, given the predictable nature of their reproductive future.^( 25 )^

Regarding correlations between gestational age and IMR, this study revealed associations between preterm (28 to 31 weeks) or post-term (more than 42 weeks) pregnancy and IMR. Kropiwiec et al.,^( 26 )^ also reported associations between higher infant mortality and gestational age of 28 to 36 weeks (odds ratio, 12.08), supporting the relation with prematurity (<37 weeks of gestation; odds ratio, 12.08), which is thought to be a relevant factor for infant mortality, particularly early neonatal mortality.

Analysis based on secondary data is a limiting factor in this study due to potential inconsistencies, such as underreporting, which persist in spite of improvements in existing data systems. However, as described by Boing et al.,^( 27 )^ it is necessary to refine data discussion to improve data systems.

## CONCLUSION

Maternal age and level of education increased among women giving birth in the Metropolitan Region of São Paulo, from 2006 to 2016. Older age and higher level of education were associated with declining infant mortality rates.
