# Supplementary material for: Infant mortality in the Metropolitan Region of São Paulo: an ecological study
Source: Einstein (Sao Paulo). 2021 Aug 2;19:eAO5663. doi: 10.31744/einstein_journal/2021AO5663 (PMC8328149; doi:10.31744/einstein_journal/2021AO5663)
Supplement: Translation

# Translation: Mortalidade infantil na Região Metropolitana de São Paulo: estudo ecológico

**Authors:** Michele Ribeiro Alexandre Nunes, Luiz Vinicius de Alcantara Sousa, Vânia Barbosa do Nascimento

**Affiliations:** 1 Brazil Centro Universitário FMABC, Santo André, SP, Brazil.

**Keywords:** Mortalidade infantil, Gravidez, Fatores de risco, Escolaridade, Idade materna

## Abstract

**Objetivo:**

Identificar a influência dos fatores de risco na mortalidade infantil da Região Metropolitana de São Paulo, segundo as características da mãe e do neonato e o tipo de parto.

**Métodos:**

Trata-se de estudo ecológico com abordagem quantitativa utilizando dados secundários dos sistemas de mortalidade infantil e nascidos vivos nos 39 municípios da Região Metropolitana de São Paulo. Variáveis do recém-nascido e maternas foram extraídas do Departamento de Informática do Sistema Único de Saúde, tendo sido apresentadas as frequências absoluta e relativa, bem como a regressão linear e o coeficiente de correlação de Pearson.

**Resultados:**

No decênio, registraram-se perfil materno com escolaridade entre 8 e 11 anos (β=73,58; p=0,023) e idade materna entre 30 e 34 anos (β=١٩,٠٤; p=0,015). O parto mais evidenciado foi o cesáreo (β=39,59; p=0,009) e a duração da gestação mais apontada foi a termo (β=-14,20; p=0,324). O período pós-neonatal apresentou regressão nos óbitos comparado com as demais faixas etárias (β=-25,30; p<0,001). Ainda, mulheres no período gestacional consideradas pré-termo (r=0,86; p<0,001) e pós-termo (r=0,95; p<0,001) tiveram chances aumentadas na taxa de mortalidade infantil.

**Conclusão:**

A faixa etária materna e o grau de escolaridade estão aumentando nas mulheres que tiveram filhos na Região Metropolitana de São Paulo, no período de 2006 a 2016. Isso também demonstra relevância na redução da taxa de mortalidade infantil.

## INTRODUÇÃO

A análise da taxa de mortalidade infantil (TMI) e de seus elementos coopera para o entendimento das condições de vida ao longo do primeiro ano, pois os motivos e as condições de risco para o óbito infantil variam ao longo do período pré-natal, do parto e do puerpério. A TMI é segmentada em períodos: a taxa de mortalidade neonatal, que pode ser subdividida em neonatal precoce e tardia, e a taxa de mortalidade pós-neonatal.^( 1 )^

Diversos aspectos estão relacionados aos óbitos infantis, sendo estes riscos socioeconômicos, comportamentais e biológicos.^( 2 )^A mortalidade neonatal é vulnerável às condições intrínsecas ou biológicas pertinentes à gestação e ao parto e relacionadas a problemas genéticos, malformação fetal e complicações no parto e no pós-parto, sendo as ações na prevenção mais complexas.^( 3 )^

Além disso, é um indicador sugestionável aos fatores externos que influenciam na ocorrência de óbitos nessa faixa etária. Corresponde às características maternas e às condições de vida, como meio ambiente, nutricionais, socioeconômicas, educação, acesso aos serviços de saúde e de bem-estar, em que essa população está inserida.^( 4 )^

Por conseguinte, as causas de mortalidade infantil podem indicar que ações preventivas conhecidas não estão sendo viabilizadas de maneira adequada.^( 5 )^

No Brasil, nas últimas décadas, ocorreu importante redução na mortalidade infantil tardia, além de atenuação da mortalidade neonatal e acréscimo dos nascimentos pré-termo.^( 6 )^

O Instituto Brasileiro de Geografia e Estatística (IBGE), por meio do censo 2010, aponta que, no período de 2000 a 2010, os óbitos de crianças menores de 1 ano tiveram declínio de 47,6% na TMI no Brasil.^( 7 )^

A pactuação diante dos Objetivos de Desenvolvimento do Milênio (ODM) leva em consideração a redução da mortalidade infantil. O Brasil, por sua vez, vem obtendo evoluções positivas na redução da mortalidade infantil, mas, considerando tal pactuação, precisa reduzir ainda mais os óbitos antes de 2030.^( 8 )^

A Região Metropolitana de São Paulo (RMSP) é considerada uma importante região brasileira por ter alta concentração populacional, desigualdade social e complexidades urbanas. Assim, políticas públicas vêm sendo implantadas nas últimas décadas, especialmente de saúde, que necessitam de informações e indicadores para orientar os programas e as ações de melhoria da qualidade de vida. Caracterizar o perfil da mortalidade infantil pode oferecer importante subsídio para acompanhamento e avaliação de tais políticas governamentais.

No Brasil, o aperfeiçoamento dos sistemas de informações em saúde do país, especialmente do Sistema de Informações sobre Mortalidade (SIM) e do Sistema de Informações sobre Nascidos Vivos (SINASC), tem contribuído para uma melhor qualidade das informações e a disseminação delas abordando a mortalidade infantil e seus determinantes.^( 9 )^

## OBJETIVO

Identificar a influência dos fatores de risco na mortalidade infantil da Região Metropolitana de São Paulo, segundo as características da mãe e do neonato e o tipo de parto.

## MÉTODOS

### Delineamento do estudo

Estudo observacional, de caráter ecológico, com abordagem quantitativa, utilizando dados secundários sobre mortalidade infantil.^( 10 )^

O período escolhido para análise das informações obtidas da mortalidade infantil foi de 2006 a 2016.

### Local de estudo

Os 39 municípios da RMSP estão localizados em torno da cidade de São Paulo (SP), na Região Sudeste brasileira. Possuem a maior concentração urbana do Brasil.^( 11 )^

Essa região foi criada em 1973 e reestruturada em 2011, pela lei complementar 1.13916, de junho de 2011, que a organizou em sub-regiões, conforme dados fornecidos pelo site (https://www.pdui.sp.gov.br/rmsp/?page_id=56).^( 12 )^Além do município de São Paulo, as sub-regiões são: Norte (Caieiras, Cajamar, Francisco Morato, Franco da Rocha e Mairiporã), Leste (Arujá, Biritiba-Mirim, Ferraz de Vasconcelos, Guararema, Guarulhos, Itaquaquecetuba, Mogi das Cruzes, Poá, Salesópolis, Santa Isabel e Suzano), Sudeste (Diadema, Mauá, Ribeirão Pires, Rio Grande da Serra, Santo André, São Bernardo do Campo e São Caetano do Sul), Sudoeste (Cotia, Embu das Artes, Embu-Guaçu, Itapecerica da Serra, Juquitiba, São Lourenço da Serra, Taboão da Serra e Vargem Grande Paulista) e Oeste (Barueri, Carapicuíba, Itapevi, Jandira, Osasco, Pirapora do Bom Jesus e Santana do Parnaíba).

Existem, nessa região, aproximadamente 21,6 milhões de habitantes, segundo estimativa do IBGE para 2018.

### Coleta de dados

O levantamento dos dados foi realizado por intermédio do *site* do Departamento de Informática do Sistema Único de Saúde (DATASUS), utilizando a ferramenta denominada TABNET, que disponibiliza informações sobre a saúde brasileira.

Os sistemas do DATASUS que foram usados para a coleta foram o SINASC e o SIM.^( 13 )^O SINASC foi instituído em 1990 pelo Ministério da Saúde, a fim de fornecer um registro sistemático de todos os nascidos vivos do país, a partir de informações oriundas da Declaração de Nascido Vivo, que abrange dados sobre a mãe, o pré-natal, o parto e o recém-nascido.^( 14 )^O SIM foi instituído em 1975 e tem como base a captação dos dados de óbitos de todo o país, provenientes da declaração de óbito, que é padronizada.^( 15 )^

A análise dos nascidos vivos seguiu agrupamentos do SINASC relativos a características da mãe, do neonato e da gestação. Da mãe, as características foram idade (agrupada nas seguintes faixas: 20 a 24; 25 a 29; 30 a 34; 35 a 39; 40 a 44; 45 a 49 e 50 a 54), tipo de parto (cesáreo ou vaginal), escolaridade (dividida em 1 a 3 anos de estudo; 4 a 7 anos de estudo; 8 a 11 anos estudo e 12 anos ou mais anos de estudo) e idade gestacional (menos de 22; 22 a 27; 28 a 31; 33 a 36; 37 a 41 e 42 semanas ou mais). Dos neonatos, foi pesquisado o sexo (masculino e feminino), e foram incluídos todos os óbitos estimados para a RMSP ocorridos em crianças menores de 1 ano, no período de 2006 a 2016.

Já no SIM, foram observadas Características relacionadas à mãe, como idade da mãe (20 a 24; 25 a 29; 30 a 34; 35 a 39; 40 a 44; 45 a 49 e 50 a 54); tipo de parto (cesáreo e vaginal); escolaridade da mãe (1 a 3 anos; 4 a 7 anos; 8 a 11 anos e 12 anos e mais) e idade gestacional (menos de 22; 22 a 27; 28 a 31; 33 a 36; 37 a 41, e 42 semanas ou mais). As características dos óbitos registradas foram faixa etária (zero a 6 dias; 7 a 27 dias e 28 a 364 dias) e sexo (masculino e feminino).

As taxas de mortalidade neonatal precoce (óbitos de zero a 6 dias de vida), neonatal (óbitos de 7 a 27 dias de vida), pós-natal (óbitos de 28 a 364 dias de vida) e infantil (óbitos menores de 1 ano de vida) foram calculadas por 1.000 nascidos vivos.

### Análise estatística

A tendência da mortalidade infantil foi verificada por meio de modelos de regressão linear, apresentando a TMI (variável dependente) e o tempo (variável independente), expressos nos anos que compõem o período de estudo (2006 a 2016).

Considerou-se nível de confiança de 95% e utilizou-se o programa estatístico Stata, versão 11.0^®^.

### Comitê de ética

Este estudo é baseado em dados secundários, não sendo capaz de identificar o indivíduo. Os dados estão disponíveis na Internet de forma livre e irrestrita, não havendo necessidade do envio deste projeto ao Comitê de Ética em Pesquisa para apreciação, segundo expresso na resolução 466/2012.

## RESULTADOS

Ao analisar as características maternas de todos os nascimentos ocorridos na RMSP, no período de 2006 a 2016, a tabela 1 mostrou que a escolaridade da mãe apresentou aumento significante de anos estudados, com ênfase para o aumento dos estratos de 8 e 11 anos de estudos (β=73,58; p=0,023) e mais de 12 anos de estudo (β=15,33, p=0,024).

**Tabela 1:** Análise das características maternas (relacionadas às parturientes) da Região Metropolitana de São Paulo

Variável	β	r	Valor de p*
Escolaridade da mãe, anos			
1-3	-10,66	0,79	<0,001
4-7	-22,30	0,38	0,025
8-11	73,58	0,39	0,023
12 ou mais	15,33	0,38	0,024
Idade da mãe, anos			
10-14	-0,07	-0,10	0,89
15-19	0,87	-0,10	0,89
20-24	-6,12	-0,05	0,495
25-29	8,03	0,01	0,301
30-34	19,04	0,43	0,015
35-39	16,26	0,71	<0,001
40-44	5,47	0,43	0,016
45-49	0,836	0,37	0,026
50-54	-0,006	-0,16	0,912
Tipo de parto			
Vaginal	-12,27	-0,03	0,437
Cesáreo	39,59	0,49	0,009
Duração da gestação, semanas			
<22	8,92	0,54	0,005
22-27	11,2	0,19	0,097
28-31	-12,24	0,68	<0,001
33-36	-7,25	0,06	0,229
37-41	14,20	0,01	0,323
42 ou mais	-3,45	0,69	0,001

Fonte: Brasil. Ministério da Saúde. Departamento de Informática do Sistema Único de Saúde do Brasil (DATASUS).

SIM-Sistema de Informações de Mortalidade. Brasília (DF): DATASUS; 2008 [citado 2020 Abr 6]. Disponível em: http://www2.datasus.gov.br/DATASUS/index.php?area=060701 ^(16)^

* regressão linear.

β: declive de regressão; r: capacidade preditiva.

Os dados revelam que as faixas etárias de mães entre 30 e 34 (β=19,04; p=0,015) e 35 e 39 (β=16,26; p≤0,001) foram as mais predominantes ao longo dos anos. Sobre o tipo de parto, o cesáreo (β=39,59; p=0,009) teve aumento com relação ao vaginal (β=-12,27; p=0,437), e, em relação à duração da gestação, partos considerados a termo (37 a 41) tiveram acréscimo na comparação com os demais (β=14,20; p=0,324).

A figura 1 apresenta redução da mortalidade infantil segundo as faixas etárias na RMSP nos anos de 2006 a 2016. Na tabela 2 , a mortalidade pós-neonatal de 28 a 364 dias apresenta significante redução na incidência de óbitos (β=-25,30; p<0,001), seguida do período neonatal precoce (zero a 6 dias), que também expressou declínio (β=-17,60; p=0,004) no total dos óbitos. Em relação ao sexo dos óbitos, os dados não revelaram significância.

**Figura 1:** Tendência temporal da taxa de mortalidade infantil

**Tabela 2:** Análise das características relacionadas aos óbitos infantis de neonatos da Região Metropolitana de São Paulo

Variável	β	r	Valor de p*
Faixa etária, dias			
0-6	-17,60	0,56	0,004
7-27	-13,62	0,71	<0,001
28-364	-25,30	0,76	<0,001
Sexo			
Masculino	321,15	0,07	0,207
Feminino	341,63	0,13	0,144

Fonte: Brasil. Ministério da Saúde. Departamento de Informática do Sistema Único de Saúde do Brasil (DATASUS).

SIM-Sistema de Informações de Mortalidade. Brasília (DF): DATASUS; 2008 [citado 2020 Abr 6]. Disponível em: http://www2.datasus.gov.br/DATASUS/index.php?area=060701 ^(16)^

* Regressão linear.

β: declive de regressão; r: capacidade preditiva.

A figura 2 apresenta a tendência dos óbitos infantis segundo a idade materna. No decênio, houve tendência de queda em sua totalidade, porém a maior queda ocorreu no ano de 2007 em todas as idades. O ano de 2008 teve aumento considerável na tendência de óbitos na faixa de etária de 20 a 24 anos, e, no ano de 2013, na faixa etária de 30 a 34 anos, vê-se tendência a novo aumento.

**Figura 2:** Tendência dos óbitos infantis segundo a idade materna

Na tabela 3 , observa-se que mulheres com mais anos de estudo – entre 8 a 11 anos (r=0,97; p<0,001) ou 12 anos ou mais (r=0,91; p<0,001) – apresentaram elevada taxa de parto cesáreo, assim como mulheres com idade entre 30 a 34 anos e 35 a 39 anos (r=0,95; p<0,001 e r=0,92; p<0,001, respectivamente). Parto vaginal prevaleceu naquelas mulheres com poucos anos de estudo (4 a 7 anos; r=0,85; p<0,001) e idade entre 20 a 24 anos (r=0,98; p<0,001).

**Tabela 3:** Correlação entre a escolaridade e idade da mãe com o tipo de parto na Região Metropolitana de São Paulo

Variável	Vaginal		Cesáreo	
	r*	Valor de p*	r*	Valor de p*
Escolaridade da mãe, anos				
1-3	0,46	0,146	0,54	0,080
4-7	0,85	<0,001	-0,08	0,810
8-11	0,45	0,159	0,97	<0,001
12 ou mais	0,48	0,128	0,91	<0,001
Faixa etária da mãe, anos				
10-14	0,58	0,059	0,42	0,191
15-19	0,85	<0,001	0,66	0,025
20-24	0,98	<0,001	0,35	0,278
25-29	0,74	0,008	0,85	<0,001
30-34	0,38	0,239	0,95	<0,001
35-39	0,21	0,528	0,92	<0,001
40-44	0,30	0,357	0,85	<0,001
45-49	-0,23	0,491	0,46	0,147
50-54	-0,71	0,045	-0,24	0,555

Fonte: Brasil. Ministério da Saúde. Departamento de Informática do Sistema Único de Saúde do Brasil (DATASUS).

SIM–Sistema de Informações de Mortalidade. Brasília (DF): DATASUS; 2008 [citado 2020 Abr 6]. Disponível em: http://www2.datasus.gov.br/DATASUS/index.php?area=060701 ^(16)^

* Teste de correlação de Pearson.

A figura 3 apresenta a tendência dos óbitos infantis segundo os anos de escolaridade materna na RMSP entre os anos de 2006 e 2016.

**Figura 3:** Tendência dos óbitos infantis segundo os anos de escolaridade materna

Na tabela 4 , destaca-se que mulheres no período gestacional considerado pré-termo, entre ٢٨ e ٣١ semanas (r=٠,٨٦; p<٠,٠٠١), e pós-termo, de 42 ou mais semanas (r=0,95; p<0,001), tiveram incidência aumentada de mortalidade infantil. Em relação à idade materna, os dados de maior significância demonstram que na faixa etária entre 35 e 39 anos (r=-0,81; p<0,002) teve redução da TMI no período estudado. A escolaridade da mãe apresentou incidência aumentada de mortalidade infantil dos estratos de 1 a 3 anos de estudos (r=0,89; p<0,001).

**Tabela 4:** Duração da gestação, idade materna e escolaridade materna, segundo taxa de mortalidade infantil na Região Metropolitana de São Paulo

Variável	Taxa de mortalidade infantil		
		r	Valor de p*
Duração da gestação, semanas			
22-27	-0,46		0,149
28-31	0,86		<0,001
32-36	0,38		0,244
37-41	-0,41		0,205
42 ou mais	0,95		<0,001
Idade da mãe, anos			
10-14	0,14		0,661
15-19	-0,10		0,757
20-24	0,13		0,696
25-29	-0,43		0,183
30-34	-0,65		0,027
35-39	-0,81		0,002
40-44	-0,55		0,077
45-49	-0,43		0,185
50-54	0,08		0,836
Escolaridade da mãe, anos			
1-3	0,89		<0,001
4-7	0,56		0,067
8-11	-0,66		0,024
12 ou mais	-0,67		0,022

Fonte: Brasil. Ministério da Saúde. Departamento de Informática do Sistema Único de Saúde do Brasil (DATASUS).

SIM-Sistema de Informações de Mortalidade. Brasília (DF): DATASUS; 2008 [citado 2020 Abr 6]. Disponível em: http://www2.datasus.gov.br/DATASUS/index.php?area=060701 ^(16)^

* Teste de correlação de Pearson.

## DISCUSSÃO

Neste estudo, considerando os partos ocorridos na RMSP, o perfil e as características maternas revelaram que as mulheres dessa região possuíam nível de escolaridade elevado (acima de 8 anos de estudo) e eram predominantemente da faixa etária entre 30 a 39 anos, com destaque para a elevação dos partos considerados a termo e o tipo de parto cesáreo como dominante. Entretanto, as condições de baixa escolaridade materna (1 a 3 anos de estudo) têm chamado atenção negativamente para óbitos infantis, pois essa variável tem sido utilizada como indicador da condição socioeconômica da mãe e de sua família, relacionando-se à qualidade dos cuidados com a saúde da criança.^( 17 , 18 )^A escolaridade materna influencia na mortalidade infantil, há relação direta entre mães sem nenhum grau de instrução e aumento no risco da mortalidade infantil.^( 17 , 18 )^Ainda, há registros de que, nos anos de 2001 a 2011 (risco relativo de 4,89 e 5,06, respectivamente) e em 2000 a 2003, mulheres com 3 anos de estudo ou menos tiveram 1,56 mais chance de serem mães.

No estudo da Região Metropolitana de Porto Alegre realizado nos de 1998 a 2006, a escolaridade materna teve bons impactos sobre as taxas de mortalidade infantil, pois a influência das mulheres com 8 ou mais anos de instrução aumentou de 46,09%, em 1996, para 60,98%, em 2008.^( 19 )^

No entanto, outros estudos comprovam que 43,2% das mães com grau de escolaridade elevado (superior a 8 anos de estudo) tiveram mais associação com o óbito infantil.^( 20 )^

Os dados da pesquisa demonstraram que a idade da mãe entre 35 e 39 anos sobressaiu como fator protetor da ocorrência da mortalidade infantil. Os achados de Alberto et al.,^( 21 )^identificaram que, em Moçambique, a idade materna mais elevada também é um fator protetor, e as mães adolescentes foram indicativo para mortalidade infantil. No Brasil e em Moçambique, os resultados indicam que mães consideradas maduras conduzem melhor a gestação, pois aderem ao pré-natal, e desempenham melhor os cuidados com a criança.

Estudo realizado em Londrina (PR) nos anos de 2000 a 2009 evidenciou que, em quase todos os biênios, os óbitos infantis foram mais frequentes entre mulheres na faixa etária dos 10 aos 19 anos. Ainda, a TMI entre adolescentes foi maior se comparada à de mães com idade reprodutiva avançada.^( 22 )^

A prematuridade é o fator de risco de mortalidade infantil mais evidenciado na literatura. Neste estudo, a maioria dos óbitos infantis aconteceu no período gestacional de 28 a 31 semanas, em consonância com o encontrado por Sanders et al.,^( 23 )^que demonstraram que a maioria das mães que concebeu seus filhos com menos de 37 semanas teve mais chances de óbitos infantis (intervalo de confiança de 6,3-66,8; p<0,001).

Em relação às características relacionadas aos óbitos infantis, este estudo destaca que houve significante diminuição nos óbitos de neonatos acima de 28 dias. Sabe-se que o principal componente da mortalidade infantil é o neonatal precoce (57,1%), com ênfase nas primeiras 24 horas de vida.^( 8 )^

Na correlação entre tipo de parto e anos de estudos da mãe, esta pesquisa mostra que aquelas com mais de 12 anos de estudo realizaram prioritariamente parto cesáreo, concordando com os achados de Riscado et al.,^( 24 )^que ressaltam que mulheres com nível socioeconômico e escolaridade elevados e usuárias do setor privado são as que mais realizam cesarianas, sendo tal conduta influenciada pela lógica do mercado na assistência ao parto.

É importante sensibilizar os profissionais de saúde para o entendimento das vias de partos, bem como dos riscos da cesariana eletiva e da prematuridade iatrogênica.^( 5 )^O parto cesáreo pode se apresentar como fator de proteção para óbito infantil, principalmente em gestações de alto risco.^( 23 )^

A relação entre via de parto e idade materna é assunto que gera bastante indagação, pois existe a preocupação de que as adolescentes não estejam preparadas para serem submetidas ao parto vaginal, pela imaturidade corporal para a parturição e por fragilidade emotiva. No entanto, o desenvolvimento obstétrico das adolescentes é similar ao das mulheres adultas quanto à via de parto. Ainda, os índices de cesariana estão aumentando com o avançar da idade materna, pois muitas mulheres mais maduras, que iniciaram a prática reprodutiva tardiamente e têm gestações planejadas, têm indicação de cesariana eletiva, por apresentarem futuro reprodutivo previsível.^( 25 )^

Quanto à correlação entre a duração da gravidez com a TMI, esta pesquisa observou que gestações classificadas como pré-termo (28 e 31 semanas) e pós-termo (com 42 semanas ou mais) tiveram aumento da ocorrência de mortes infantis. Kropiwiec et al.,^( 26 )^também registraram o aumento da mortalidade infantil para gestações entre 28 a 36 semanas de gestação (razão de chance de 12,08), justificando a relação com a prematuridade (<37 semanas de gestação; razão de chance de 12,08), associação esta entendida como fator relevante para o óbito infantil – especialmente o neonatal precoce.

A análise de informações obtidas de dados secundários gera limitações no estudo, pois, apesar de melhorias nos sistemas de informações, ainda existem inconsistências, como subnotificações. No entanto, como descreve Boing et al.,^( 27 )^é necessário aprimorar a discussão dos dados para melhorar o sistema de informação.

## CONCLUSÃO

A faixa etária das mães e o grau de escolaridade aumentaram nas mulheres que tiveram filhos na Região Metropolitana de São Paulo, no período de 2006 a 2016. Ainda, ambas, a faixa etária e a escolaridade aumentadas mostraram-se relevantes na redução da taxa de mortalidade infantil.
